# Imaging features of sublingual dermoid cysts: a report of four cases

**DOI:** 10.1016/j.radcr.2022.05.025

**Published:** 2022-06-13

**Authors:** Peruze Celenk, Cetin Celenk, Husniye Demirturk Kocasarac

**Affiliations:** a Department of Oral and Maxillofacial Radiology, School of Dentistry, Ondokuzmayis University, Samsun, Turkiye; b Department of Radiology, Faculty of Medicine, Ondokuzmayis University, Samsun, Turkiye; c Division of Oral and Maxillofacial Radiology, Department of General Dental Sciences, Marquette University School of Dentistry, Milwaukee, WI, USA

**Keywords:** Dermoid, Sublingual, Imaging, MRI, Diffusion

## Abstract

Dermoid cysts of the floor of the mouth are rare lesions presumed to be caused by entrapment of germinal epithelium during the closure of the mandibular and hyoid branchial arches. They usually manifest as nonpainful swelling. Developmental cysts are histopathologically classified into 3 types: epidermoid, dermoid, and teratoid. An ultrasound scan is commonly used as the first choice to investigate a lesion. Other imaging methods, such as the US, CT, and MRI, are used for differential diagnosis. This article's aim is to present the imaging findings of 4 cases of sublingual dermoid cysts and to review the literature.

## Introduction

Developmental cysts are histopathologically classified into 3 types: epidermoid, dermoid, and teratoid. Dermoid cysts occur most frequently in patients aged 15-35 years, but they are observed in all age groups.

About 10% or less of dermoid cysts occur in the head and neck region, and only 1%-2% develop in the oral cavity. About 25% of dermoid cysts in the oral cavity occur on the floor of the mouth [1,2].

Dermoid cysts present as soft and painless slow-growing masses at the sublingual, submental, and submandibular regions, with possible associated dyspnea and disorders of swallowing, chewing, and speaking [1], [2], [3].

An ultrasound scan is commonly used as the first choice to investigate the lesion [4]. This report presents 4 cases of dermoid cysts on the floor of the mouth, and a review of requirements for diagnosis was conducted.

## Case 1

**Fig. 1:**
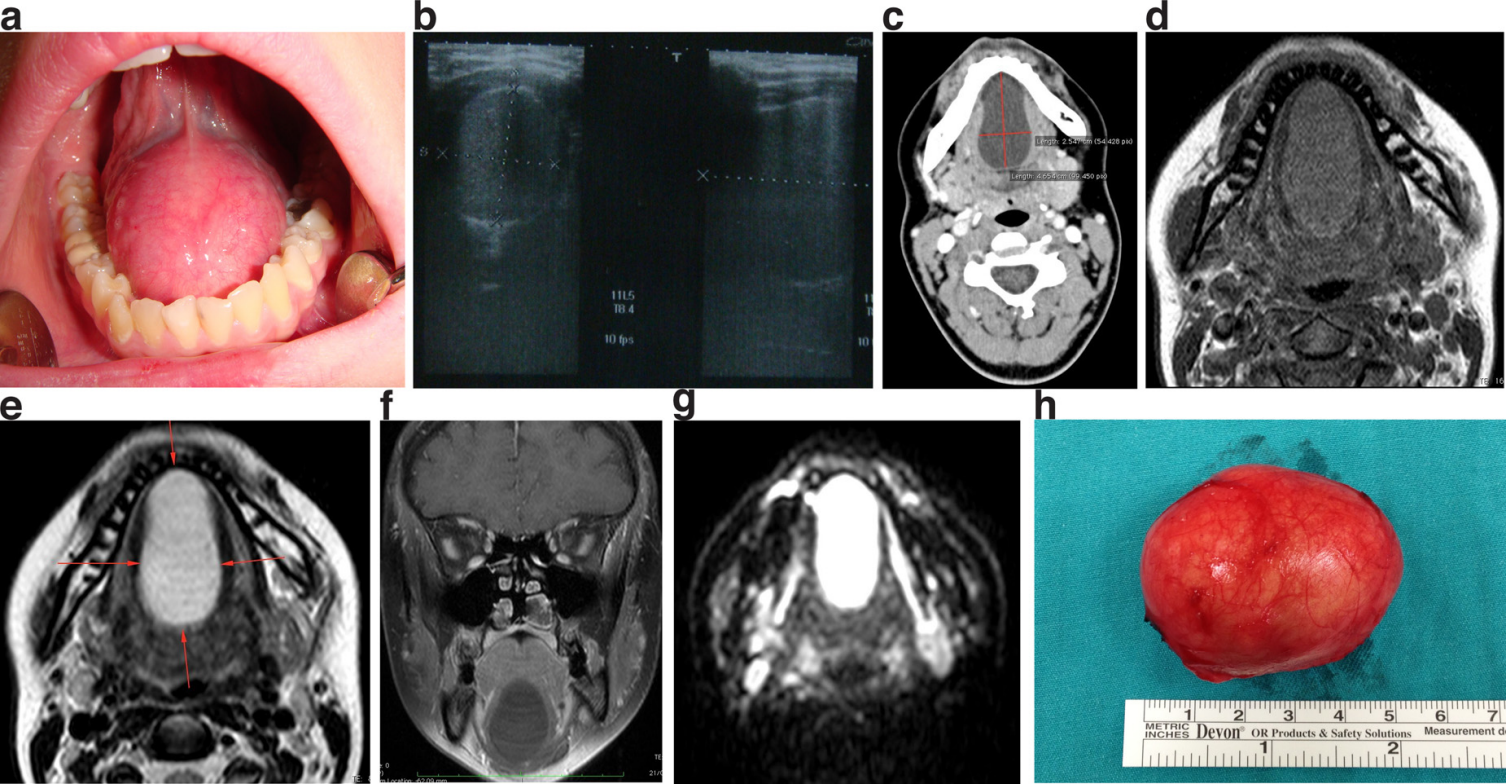
Imaging of 18-year-old female patient with sublingual dermoid cyst. (A) Prominent midline swelling in the floor of the mouth during inspection; (B) well-circumscribed and hypoechoic in ultrasonography; (C) well-circumscribed and hypodense in CT; well-circumscribed in MRI; (D) hypointense in T1WI and (E) hyperintense in T2WI; (F) no enhancement in T1W+C; (G) diffusion restricted in DWI; (H) well-circumscribed excised specimen.

An 18-year-old healthy female presented with a toothache. Intraoral examination revealed a large, soft, fluctuant, and solitary swelling in the midline of the floor of the mouth (Fig. 1A). Although the tumor was large, the patient had no complaints. Ultrasonography (US), computed tomography (CT), and magnetic resonance imaging (MRI) showed a well-defined homogenous cystic lesion on the floor of the mouth. In the US, the mass was shown to be hypoechoic (Fig. 1B). In the CT, it demonstrated hypodensity (Fig. 1C). In the MRI, it was hypointense in T1WI (Fig. 1D) and hyperintense in T2WI (Fig. 1E), and it had no enhancement in T1W+C (Fig. 1F). Diffusion-weighted imaging (DWI) showed diffusion restriction (Fig. 1G). The cystic lesion measured 24 × 40 × 48 mm. Based on the imaging findings, a diagnosis of the dermoid cyst was suggested. The cyst was removed under general anesthesia at the otorhinolaryngology clinic (Fig. 1H). The diagnosis of the dermoid cyst was made histopathologically. Microscopic examination revealed stratified squamous epithelium, sebaceous glands in the cyst wall, and keratin fibers in the lumen. No carcinomatous changes could be identified. Pathology confirmed the diagnosis of a dermoid cyst. The postoperative course was uneventful, and there was no evidence of recurrence 1 year after surgery.

## Case 2

**Fig. 2:**
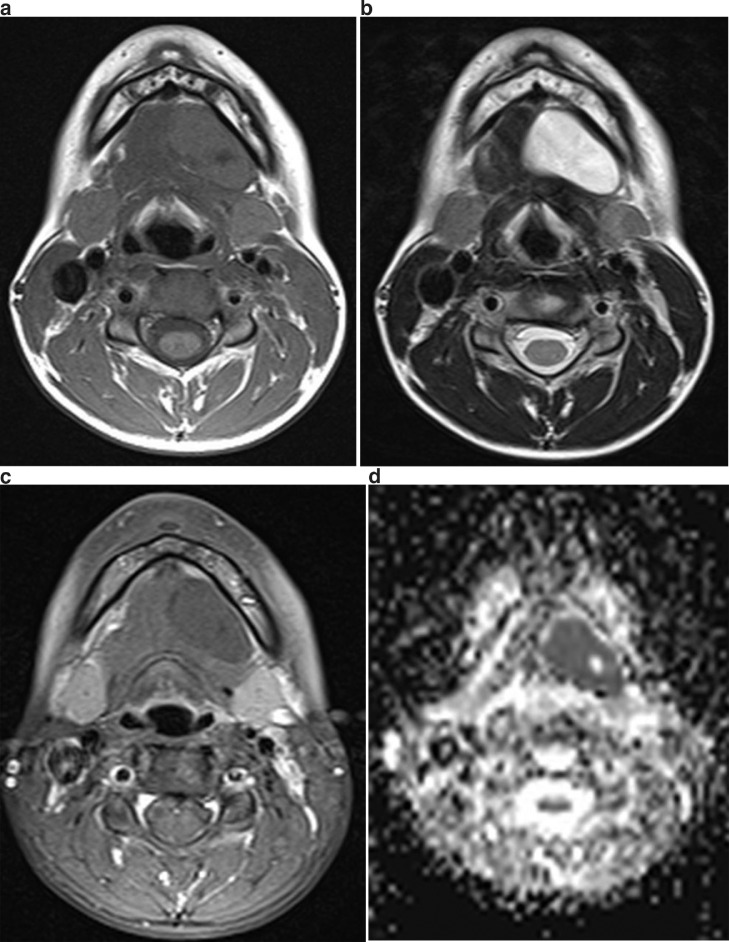
Imaging of 12-year-old female patient with dermoid cyst on left of floor of mouth. Well-circumscribed in MRI; (A) hypointense in T1WI and (B) hyperintense in T2WI; (C) no enhancement in T1W+C, (D) extremely low SI in ADC.

A 12-year-old healthy female presented complaining of swelling involving both submental and sublingual areas evolving since birth. Although the tumor was large, she did not complain of anything except swelling. Intraoral examination revealed a large, soft, fluctuant, and solitary swelling on the left of the floor of the mouth. MRI showed hypointensity in T1WI (Fig. 2A), hyperintensity in T2WI (Fig. 2B), no enhancement in STIR+C (Fig. 2C), and extremely low signal intensity (SI) on the apparent diffusion coefficient (ADC) map, which indicated marked restriction (Fig. 2D). The lesion was approximately 33 × 17 × 29 mm. Based on the imaging findings, the diagnosis of a dermoid cyst was suggested. The cyst was removed under general anesthesia at the otorhinolaryngology clinic. Diagnosis of the dermoid cyst was made histopathologically.

## Case 3

**Fig. 3:**
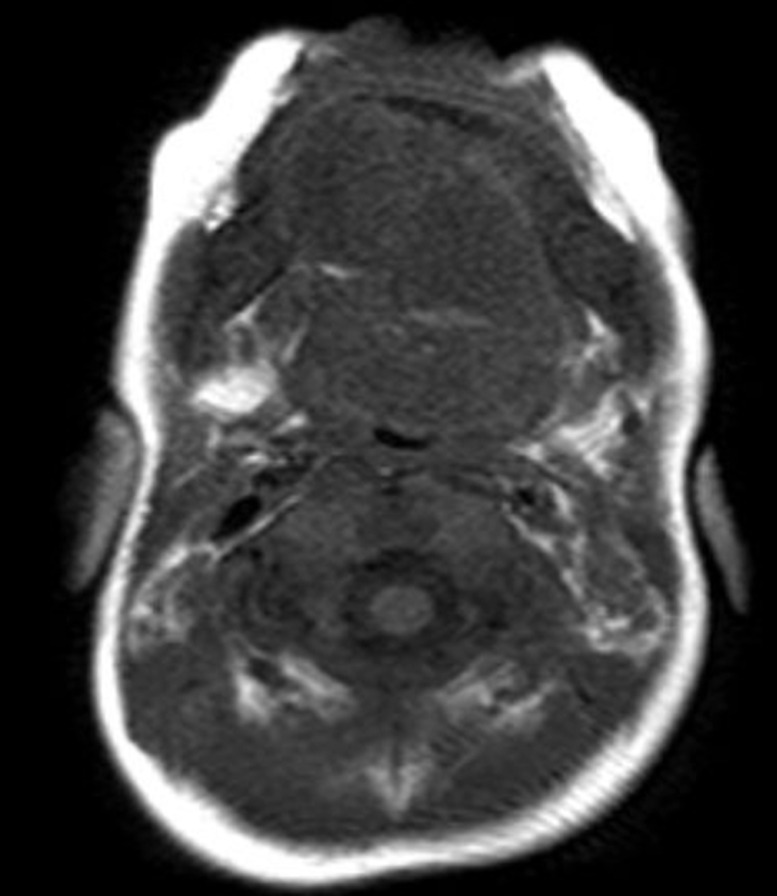
Imaging of 2-month-old female patient with sublingual dermoid cyst. In T2W, MRI showed well-defined, well-circumscribed, homogenous cystic lesions adjacent to each other, involving the right and midline of the floor of the mouth. The lesions measured approximately 15 × 14 × 12 mm and 15 × 13 × 11 mm.

A 2-month-old healthy girl presented for a submental sublingual mass present since birth. Intraoral examination revealed a large, soft, fluctuant, solitary swelling in the right and midline of the floor of the mouth. The T2W MRI (Fig. 3) showed 2 well-defined, homogenous cystic lesions adjacent to each other involving the right and midline of the floor of the mouth, measuring approximately 15 × 14 × 12 mm and 15 × 13 × 11 mm. Based on the imaging findings, a diagnosis of dermoid cysts was suggested. The cysts were removed under general anesthesia at the otorhinolaryngology clinic. Diagnosis of the dermoid cysts was made histopathologically.

## Case 4

**Fig. 4:**
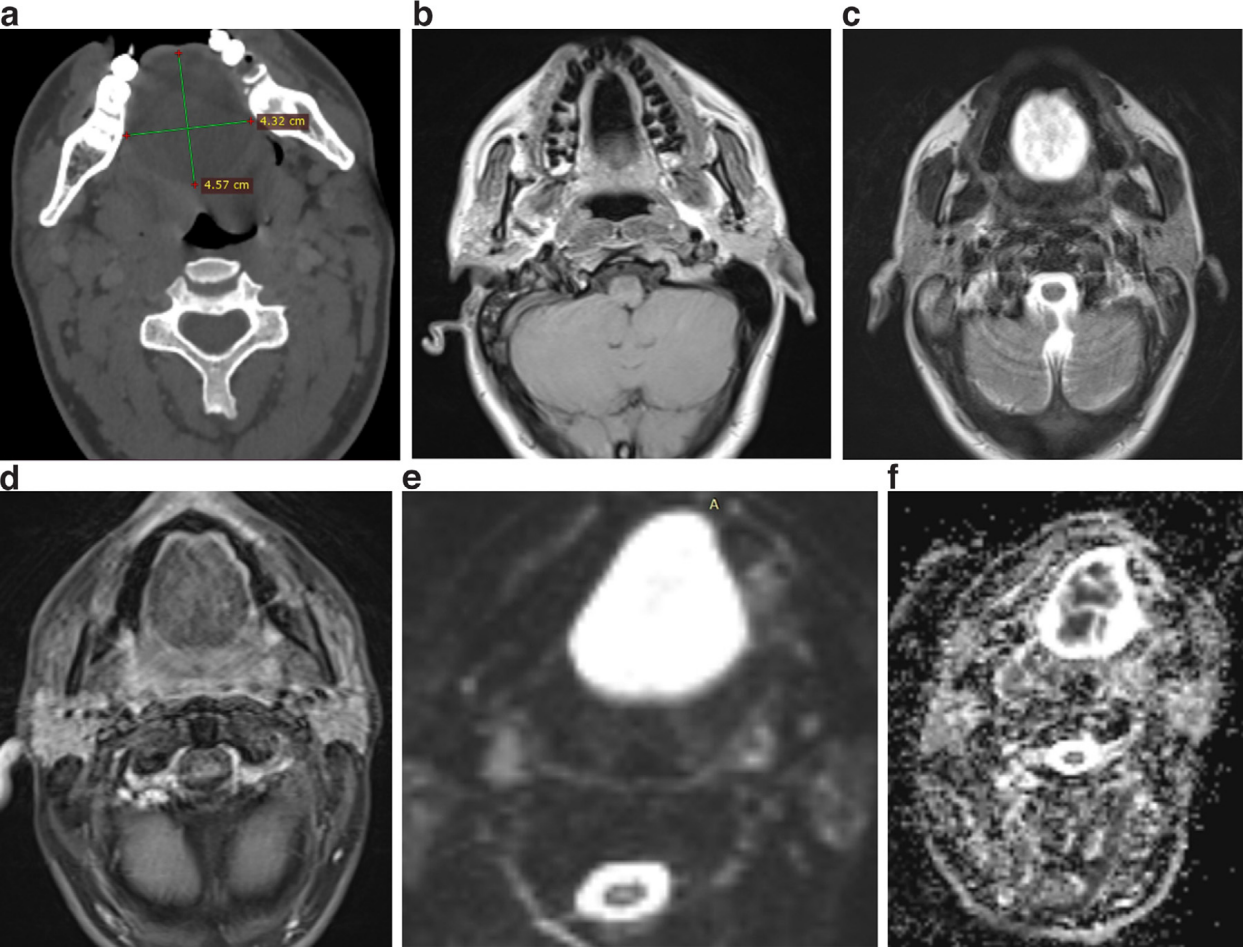
Imaging of 19-year-old female patient with sublingual dermoid cyst. Well-circumscribed in CT; (A) hypodense and well-circumscribed in MRI; (B) hypointense in T1WI; (C) hyperintense in T2WI, (D) no enhancement in STIR+C; (E) diffusion restricted in DWI; (F) extremely low SI in ADC; cystic lesion measured 41 × 45 × 65 mm.

A healthy 19-year-old girl visited our clinic with the complaint of a submental/submandibular mass that had been present since birth. Intraoral examination revealed a large, soft, fluctuant, and solitary mass. In CT, it was hypodense (Fig. 4A); in MRI, T1WI showed hypointensity (Fig. 4B), T2WI showed hyperintensity (Fig. 4C), and STIR+C was unenhanced (Fig. 4D). DWI showed diffusion restriction (Fig. 4E), and a 41 × 45 × 65 mm hypointense cystic lesion was seen. The cyst was removed under general anesthesia at the otorhinolaryngology clinic. A diagnosis of the dermoid cyst was made histopathologically.

## Discussion

Developmental cysts are histopathologically classified into 3 types: epidermoid, dermoid, and teratoid. Histologically, all dermoid is covered by the epidermis. The contents of the cyst lining determine the cyst's histological category. Epidermoid cysts have an epithelial-lined wall that may be partly keratinized, with no evidence of skin appendages. However, dermoid cysts are cysts with an epithelial lining enclosing skin appendages, such as hair, hair follicles, sebaceous glands, and sweat glands. The teratoid type is also epithelium-lined, and it contains mesodermal or endodermal elements, such as muscle, bone, teeth, and mucous membranes [2], [3], [4], [5].

A dermoid cyst is a lesion thought to be caused by entrapment of germinal epithelium during the closure of the mandibular and hyoid branchial arches. These cysts are common in lines of fusion.

Because adnexal structures were found, these 4 cases were evaluated as dermoid cysts. Because of their contents, diffusion restrictions are expected in MRI of dermoid cysts. Diffusion restriction of dermoid cysts may help in the differential diagnosis.

The diagnostic algorithms for suspected dermoid cysts should include US, CT, and/or MRI. CT and MRI allow for precise localization of the lesion in relation to anatomic structures. Complex fluid attenuation is characteristic of dermoid cysts. Homogeneous fluid attenuation is seen in epidermoid cysts [2,[4], [5], [6], [7], [8], [9]]. Fluid attenuation in epidermoid cysts and the presence of fat in dermoid cysts can help differentiate the 2. Both CT and MRI show that the cyst's thin wall usually enhances following intravenous administration of contrast material.

Sublingual, submandibular, and neck masses are considered in the differential diagnosis of dermoid/epidermoid cysts. The differential diagnosis of sublingual lesions includes oral cavity abscess, ranula, lymphatic malformation, squamous cell carcinoma nodes, and thyroglossal duct cysts [4]. Simple ranulas may mimic unilateral cystic masses. Thyroglossal duct cysts are midline cystic neck masses between the hyoid and the foramen caecum. If it is in the posterior tongue, a thyroglossal duct cyst may mimic an epidermoid cyst. Most patients are between 10 and 35 years of age. In a series of 16 cases, the mean age was 28 years, and the ratio of men to women was 3:13 [7]. Four of the 4 patients in our study were girls, and their mean age was 13.

Most cysts in the floor of the mouth occur in the midline, and lateral dermoid cysts are rarely observed [4]. One of the 5 cystic lesions in our study's 4 patients was on the right side.

Dermoid cysts’ typical characteristics include slow growth, presenting in early adult life as asymptomatic swelling that occasionally may cause elevation of the tongue, interference with speech, and the appearance of a double chin [4]. Cystic hygromas, ectopic thyroid glands, lipoma, oral cavity abscesses, tumors of the floor of the mouth, or other mass forms may confuse the diagnosis. A definitive diagnosis is provided by the histologic specimen. Malignant transformation of cysts to squamous cell carcinoma has been reported. When cysts are discovered, the early operation is desirable.

An abscess is a painful mass in the oral cavity. A rim-enhancing cystic structure is observed together with diffuse cellulitis and edema in the oral cavity. A thyroglossal duct cyst is a cystic neck mass located between the hyoid and foramen caecum and in the midline. Those located posterior to the root of the tongue are similar to epidermoid cysts.

Metastatic lymph nodes of oral cavity squamous cell carcinomas appear as cystic structures in the submandibular region. Oral cavity lymphatic malformations are trans-spatial cystic lesions. High fluid levels may be seen, resulting in hemorrhage. If they are infected, they may have proteinaceous contents.

A simple ranula may mimic an epidermoid cyst located in the sublingual space. Ranulas are usually unilateral cystic lesions. A diving ranula is a trans-spatial cornet-shaped unilocular lesion. Its tail is in the sublingual space, and its head is in the submandibular space.

Therapy for dermoid cysts consists of surgical excision. If the lesion is located under the mylohyoid muscle, it usually is removed using an external approach. If it is above the mylohyoid muscle, it should be removed using an intraoral approach. Therefore, the identification of the cyst location in relationship to the mylohyoid muscle on CT and MRI is highly important in surgical planning [2,^4^,^5^,8]. Dermoid cysts’ linings are generally very thick, and they can be easy to remove.

## Conclusion

In MRI, adnexal structures contained by dermoid cysts represent heterogeneous signal intensity in T1- and T2-weighted imaging and diffusion restriction in diffusion series. The diffusion restriction of dermoid cysts is a finding that can be easily applied in differential diagnosis and provides almost 100% accurate results.

MRI, US, and CT imaging features of a rare intraoral dermoid cyst were presented. US, CT, and MRI provide important diagnostic information in the diagnosis and differential diagnosis of dermoid cysts.

## Patient consent

The authors confirm that written and signed consent for publication was obtained from the patients in this case report.

## Ethics approval

Yes.
